# Influence of Vehicle Number on the Dynamic Characteristics of High-Speed Train-CRTS III Slab Track-Subgrade Coupled System

**DOI:** 10.3390/ma14133662

**Published:** 2021-06-30

**Authors:** Qingyuan Xu, Hao Sun, Lexuan Wang, Lei Xu, Wei Chen, Ping Lou

**Affiliations:** Department of Civil Engineering, Central South University, Changsha 410075, China; xuqingyuan1972@csu.edu.cn (Q.X.); sunhaoshine@csu.edu.cn (H.S.); wanglexuan@csu.edu.cn (L.W.); xu.lei@csu.edu.cn (L.X.); chenwei.csu@foxmail.com (W.C.)

**Keywords:** CRTS III slab track, subgrade, vehicle number, coupled dynamic, high speed train, railway

## Abstract

In this paper, a high-speed train–CRTS III slab track–subgrade coupled dynamic model is established. With the model, the influence of vehicle number on the dynamic characteristics of a train–CRTS III slab track–subgrade coupled system with smooth and random track irregularity conditions for conventional and vibration-reduction CRTS III slab tracks are theoretically studied and analyzed. Some conclusions are drawn from the results: (1) the largest dynamic responses of the coupled system for all items and cases are no longer changed when the vehicle number exceeds three, and three vehicles are adequate to guarantee the simulation precision to investigate the dynamic responses of the coupled system. (2) The acceleration of the car body has almost no relation with the vehicle number, and only one vehicle is needed to study the vehicle dynamics using the train–CRTS III slab track–subgrade coupled dynamic model. (3) For the conventional CRTS III slab track on a subgrade, the vehicle number has a negligible influence on the accelerations of the rail, slab, and concrete base, the positive and negative bending moments of the rail, the compressive force of the fastener, and the positive bending stress of slab, but it has a large influence on the tension force of the fastener, and the negative bending stresses of the slab and concrete base. Only one vehicle is needed to study track dynamics without considering the tension force of the fastener, the negative bending stresses of the slab and concrete base, otherwise, two or more vehicles are required. (4) For vibration reduction of the CRTS III slab track on a subgrade, the number of vehicles has some influence on the dynamic responses of all track components, and at least two vehicles are required to investigate the track dynamics.

## 1. Introduction

In recent years, high speed railways have been widely used around the world due to their outstanding advantages, such as their large capacity, comfort, punctuality, safety, and environmental friendliness. However, due to the dynamic impacts of high-speed trains, maintenance work for traditional ballasted tracks is difficult, and accompanied by a large consumption of labor and economic resources. To reduce maintenance work, ballastless track structures are widely used in high speed railway lines. Apart from the conventional ballastless track widely used in high speed railway, vibration-reduction ballastless tracks [1] are applied to places where a reduction in vibrations transmitted from high speed railways into the surrounding soil and nearby buildings is highly demanded.

With this background, the China railway track system (CRTS) III slab track was researched and developed by China to overcome the shortcomings of the CRTS I slab track [2], CRTS II slab track [3], and the double-block ballastless track [4]. The CRTS III slab track replaces the easily damaged CA mortar in the CRTS I and II slab tracks [5,6] with self-compacting concrete as a filling layer [7]. The CRTS III slab track has been widely used in high-speed railway lines in China since 2010 due to its high performance.

With the development of high-speed railways and ballastless tracks, the issue of the dynamics of train–ballastless track subgrades become prominent, and a great deal of research work has been done on these aspects. Lei et al. [8] developed a vehicle–CRTS II slab track-subgrade coupling dynamic model, in which a new type of slab track element was presented, and several application examples were illustrated. Zhu et al. [9] established a 3D coupled dynamic model of vehicle–CRTS II slab tracks on a subgrade to calculate the vertical and lateral rail-supporting forces, and then those forces were inputted into a 3D nonlinear finite element model to investigate the evolution of interface damage and its influence on the dynamic response of the slab track. Yang et al. [10] studied the effects of random track irregularity and vehicle velocity on the dynamic responses of slab tracks using a vehicle-CRTS I slab track in a subgrade interaction model, in which composite track elements were used to rapidly model the slab track. Later, Yang et al. [11] developed a vehicle-slab track with a subgrade coupled dynamic model in the frequency domain to investigate the response and transfer characteristics of a ballastless track. Feng et al. [12] studied the influence of the seam between a slab and CA mortar of a CRTS II slab track in terms of the vibration characteristics of the vehicle-track system using a vehicle-CRTS II slab track with a subgrade coupling dynamic model developed using ABAQUS^®^ software. Xu et al. [13] proposed a probabilistic model for simulating random track irregularities in a vehicle-slab track with a subgrade coupled dynamic model to clarify the random vibration characteristics and probabilistic relationships between random track irregularities and dynamic behaviors of vehicle-track systems. Recently, using 8-node solid elements to model the subgrade, Xu et al. [14] developed a matrix-coupled model for vehicle-slab track-subgrade interactions in a 3D space, and the accurateness, efficiency, and stability of the model were elaborated using numerical examples. Considering the influence of random track irregularity, Sun et al. [15] proposed a numerical method, in which the vehicle-CRTS I slab track with a subgrade coupled dynamic model and the Kalker’s variational method and the material wear model were combined to predict non-uniform rail wear evolution. Chen et al. [16] established a vertical model for vehicle-CRTS II slab track-subgrade dynamic interactions using the Green function method to study wheel polygonal wear. Aggestam et al. [17] developed a 3D slab track model in ABAQUS^®^ using Python scripts, then the system matrices of the model were exported to MATLAB ^®^ where the simulation of vehicle–track dynamic interactions was performed. Li et al. [18] developed a nonlinear 3D-coupled vehicle-slab track model using LS-DYNA^®^ to investigate the influences of dynamic material properties of slab track components on train–track vibration interactions. Wang et al. [19] investigated train-induced dynamic stress statistics in a subgrade surface using a vertical vehicle-track-subgrade coupled dynamic model. Xin et al. [20] established a vehicle-slab track at the transition zone of a coupled dynamic model to study reasonable transition lengths, as well as the number and stiffness coefficient of the rubber mat at the transition zone between a fixed slab track and a floating slab track. Guo et al. [21] put forward an iterative approach on the basis of vehicle–track coupled dynamics theory and an empirical model for cumulative plastic deformation of the subgrade to predict long-term track degradation of a ballastless track due to the evolution of differential subgrade settlement in a high-speed railway. Cai et al. [22] established a rigid–flexible coupling vehicle-CRTS III slab track-subgrade dynamic model to research the influence of subgrade frost heave on the dynamic behavior of a high-speed railway vehicle.

A train running on realistic rail lines consists of several vehicles. However, in References [8,9,10,11,12,13,14,15,16,17,18,19,20,21,22], only one vehicle was considered in the dynamic model. To be more consistent with actual operational situations, some scholars used train–ballastless track-subgrade-coupled dynamic models to analyze railway dynamics. Galvin et al. [23] established a general and fully 3D multibody-finite element-boundary element model to research vibrations, where a train with 10 vehicle passing on non-ballast and ballast tracks for different train speeds was considered. Kece et al. [24] developed a 2D slab track on a subgrade dynamic model to study slab track performance using the measured wheel load time history of an Amtrak Acela train with eight vehicles as the exciting force. Xu et al. [25] established a 3D train-slab track on a subgrade dynamic model, which can consider the wheel–rail separation and the vehicle formation of a train, as well as the advantages and engineering applicability of the model, were illustrated using three numerical examples. Deng et al. [26] established a wind–train-CRTS I slab track coupling dynamic model with MATLAB^®^, in which wind loads were first obtained via a computational fluid dynamics simulation, and then the wind loads were inputted into the dynamic model. The traffic safety when a CRH3 high-speed train with three vehicles passed through two types of windproof facilities under crosswind conditions were studied and compared.

As can be seen from the research above, a great deal of work has been conducted on the vehicle–ballastless track–subgrade and train–ballastless track–subgrade coupled dynamics. However, these research works still have some limitations. Firstly, the dynamic response differences for the vehicle–ballastless track–subgrade coupled dynamics and train–ballastless track–subgrade coupled dynamics are lacking in-depth, comparative study. Secondly, the applicability of the vehicle–ballastless track–subgrade coupled dynamics is not distinct for different ballastless-track structures and track irregularity conditions. Thirdly, it is not clear how many vehicles should be used to achieve a balance between the calculation precision and efficiency for a train–ballastless track–subgrade coupled model. Fourthly, most of the previous research work focused on dynamic vibration responses and dynamic wheel–rail and fastener forces of the coupled system; however, the dynamic bending stresses of the ballastless track, which are key factors to design a ballastless track, especially for the vibration-reduction ballastless tracks, were not examined in detail.

To overcome these limitations, in this paper, a high-speed train-CRTS III slab track–subgrade coupling dynamic model, which can consider both the conventional CRTS III slab track, as well as the vibration-reduction CRTS III slab track, is established. The model is verified using results calculated by the ANSYS^®^ program. With the model, the influence of the vehicle number on the dynamic characteristics of the train–CRTS III slab track–subgrade coupled system, for the conventional and vibration-reduction CRTS III slab tracks, with smooth and random track irregularity conditions, is studied and analyzed.

The novelties of this paper are as follows: Firstly, both the short and middle long wavelength random track irregularities were considered, so that the high frequency vibration of the track structure could be realistically simulated. Secondly, the influence of the vehicle number on the dynamic characteristics of the train–CRTS III slab track–subgrade coupled system for different ballastless track structures and track irregularity conditions were studied. Thirdly, in addition to the traditional dynamic vibration responses and the dynamic wheel–rail and fastener forces of the coupled system, the dynamic bending stresses of the ballastless track were also studied.

The research work can provide a theoretical basis to reasonably choose the vehicle number in modeling train–ballastless track–subgrade coupled systems with different ballastless track structures, track irregularity conditions, and research items, so that a balance between the calculation precision and efficiency could be achieved. Because of the required model length, the moving distance of the train and the simulation time can be greatly reduced with a low vehicle number in modeling the train–ballastless track–subgrade coupled system.

## 2. Train–CRTS III Slab Track–Subgrade Coupling Dynamic Model

**Figure 1:**
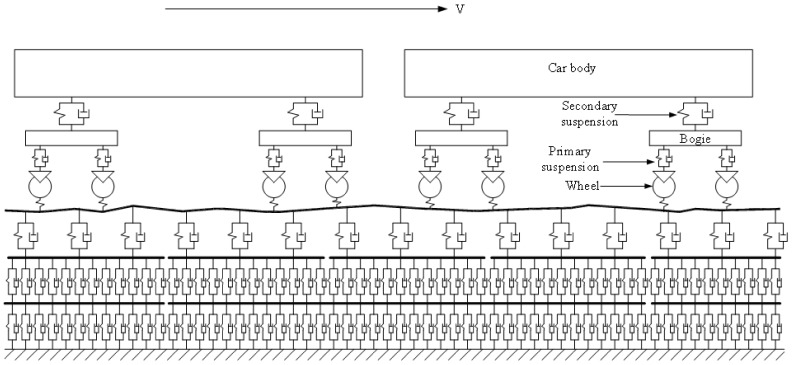
Schematic of train–CRTS III slab track–subgrade coupling dynamic model.

A dynamic model for a high-speed train traveling on a CRTS III slab track supported by a Winkler foundation at a constant speed (v) along the longitudinal direction is shown in Figure 1. The model consists of four sub-models, namely, the high-speed train sub-model, the wheel–rail interaction sub-model, the CRTS III slab track–subgrade sub-model, and the track irregularity sub-model.

### 2.1. High Speed Train Sub-Model

The train sub-model includes several identical vehicles. The vehicle number is 2 in Figure 1. Each vehicle is composed of a car body, two bogies, and four wheel-sets. 

In reality, the car body is not rigid; however, due to the excellent vibration-reduction capacity of the primary and secondary suspension systems, the car body is widely modeled as rigid body in the study of train/vehicle–ballastless track–subgrade coupled dynamics due to its acceptable calculation accuracy and simplicity.

In this paper, the car body, bogie, and wheel-set are modeled as rigid body. The primary suspension between the bogie and wheel-set, and the secondary suspension between the car body and bogie are modeled as linear spring-damper elements. The car body and bogie have bounce and pitch motions and the wheel-set only has a vertical vibration; therefore, there are 10 degrees of freedom for each vehicle. The dynamics equations for the vehicle can be found in Reference [27].

### 2.2. Wheel–Rail Interaction Sub-Model

The wheel–rail interaction sub-model is the same as the wheel–rail interaction sub-model in References [28,29]. The wheel–rail force can be calculated using the Hertzian nonlinear contact theory. For more details, References [28,29] can be consulted.

### 2.3. CRTS III Slab Track–Subgrade Sub-Model

The total length of a high-speed train with 8 vehicles is about 200 m, and the moving distance of the train was 300 m in this study to reasonably consider the long wavelength of the random track irregularity in the dynamics of the coupled system. To consider the long length and the moving distance of the train, and to achieve balance between calculation precision and efficiency at the same time, multi-scale modeling technology was used in the CRTS III slab track–subgrade sub-model. 

**Figure 2:**
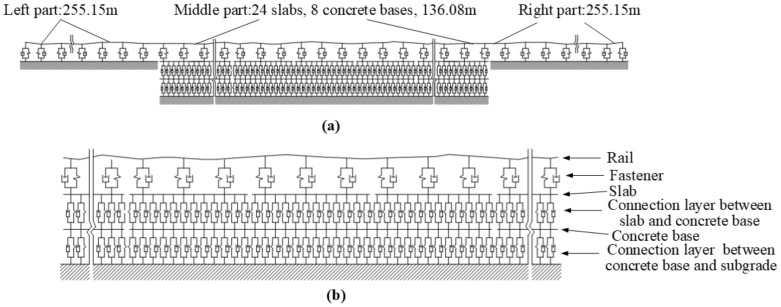
Schematic of CRTS III slab track–subgrade sub-model: (**a**) overview and (**b**) partial view.

The overview of the CRTS III slab track–subgrade sub-model, as well as a partial view of the middle part of the CRTS III slab track–subgrade sub-model, are shown in Figure 2a,b, respectively. The sub-model consists of three parts. On the left and right sides of the sub-model, only the rail and fastener are considered. In the middle part of the sub-model, a fine model is adopted. 

In the sub-model, the length of the left and right parts are 255.15 m and the length of the middle part is 136.08 m. The length of each slab is 5.67 m, the length of each concrete base is 17.01 m, and 24 slabs and 8 concrete bases are modeled in the middle part.

The rail, slab, and concrete base in the sub-model are modeled as Bernoulli–Euler linear beam elements. The fasteners, the connection layer between the slab and the concrete, as well as the layer between the concrete base and subgrade are modeled as linear spring-damper elements.

**Figure 3:**
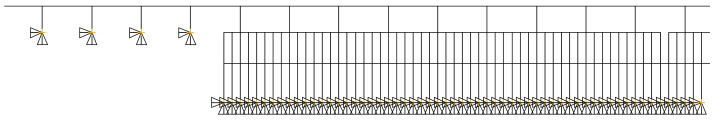
Layout of nodes and elements of the CRTS III slab track–subgrade sub-model.

In order to properly reflect the continuous support of the connection layer between the slab and concrete base, as well as between the concrete base and the subgrade, a small mesh size of 0.105 m in length is adopted. The layout of the nodes and the elements of the CRTS III slab track–subgrade sub–model for the connection between the left and central parts in Figure 2a is shown in Figure 3. There are 5692 nodes and 10,769 elements in the sub-model.

A static wheel load is applied to the central part and the left part in the sub-model, respectively, to calibrate the stiffness of the track foundation in the left and right parts. The equivalent stiffness of the track foundation is determined according to the relative error between the maximum vertical rail displacements of the central part and those of the side part and is less than 10^−6^. Detailed procedures can be found in Reference [30].

It should be mentioned that both the conventional and vibration-reduction CRTS III slab track–subgrade sub-models can be modeled by setting the different stiffnesses of connection layers between the slab and concrete base in Figure 2.

### 2.4. Track Irregularity Sub-Model

The low interference spectrum of a German high-speed railway, which is widely used as middle long wavelength random track irregularity spectrum in the train/vehicle-track–track foundation coupled dynamic model, is used to generate the random track irregularity sample of middle long wavelength.

The wavelength of the low interference spectrum of the German high-speed railway is longer than 1 m, and it cannot accurately reflect the influence of short wavelength random track irregularities on the dynamics of train–slab track–subgrade coupled system. The Sato spectrum, which is widely used in wheel–rail rolling noise [31,32], wheel–rail wear [33], and high-frequency wheel–rail contact force [34], is used to generate short wavelength random track irregularities, in which the wavelength is below 1 m.

**Figure 4:**
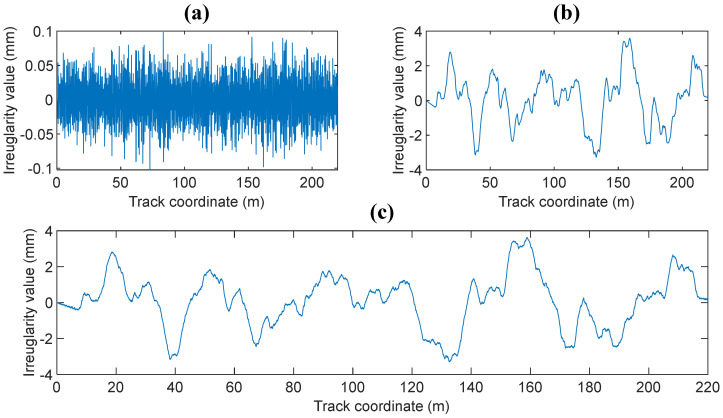
Samples of the: (**a**) short wavelength; (**b**) middle long wavelength; (**c**) combined random track irregularity.

The expression of the German low interference spectrum can be found in References [10,29], and that of the Sato spectrum can be found in Reference [29]. The generated samples according to the Sato spectrum and the low interference spectrum of the German high-speed railway are shown in Figure 4a,b respectively. The combined samples, which are generated by superimposing these two samples, are shown in Figure 4c.

## 3. Establishment and Solution of Train–CRTS III Slab Track–Subgrade Coupled System

Using the principle of total potential energy using the stationary value in elastic system dynamics presented by Zeng [35,36], the dynamic equations of motion for the train–CRTS III slab track–subgrade coupling system can be derived. 

To solve the dynamics equations of the train–CRTS III slab track–subgrade coupling dynamic model, the solution of the static model is determined first. Then, the static equilibrium position is taken as the initial condition for the subsequently dynamic analysis. Finally, the dynamic analysis for a moving train is carried out at each time step.

## 4. Calculation Parameters

The first wheel-set of the CRH3 high-speed train is located at the beginning of the 5th slab at the initial time; the train speed is 300 km/h and the time step is 6 × 10^−5^ s. The moving distance of the train is 300 m and 60,000 time steps are used to simulate the moving train in each case. The other detailed parameters for the train are listed in Table 1. The parameters of the CRTS III slab track–subgrade sub-model are listed in Table 2.

## 5. Verification of the Coupled System

The computer programs for the high speed train–CRTS III slab track–subgrade coupled system are, respectively, developed in MATLAB (MATLAB R2018a, MathWorks, Natick, Massachusetts, USA)^®^ and ANSYS^®^ (Canonsburg, PA, USA) platform.

In Reference [29], the calculation results calculated using the program developed in MATLAB^®^ were verified by measured in situ data from the Shiziyang tunnel of the Guangzhou–Hong Kong high-speed railway line in China. The program was further verified by the calculation results using the program developed in the ANSYS^®^ platform.

The high-speed train sub-model, the wheel–rail interaction sub-model, the CRTS III slab track–subgrade sub-model, and the track irregularity sub-model in the ANSYS^®^ platform are the same as those in MATLAB^®^ and are described in detail in Section 2.

In ANSYS^®^, the rail, slab, and concrete base are modeled as BEAM3 linear beam elements. The fasteners, the connections between the slab and concrete base, the connections between the concrete base and the subgrade are modeled as linear COMBIN14 spring-damper elements. The detailed procedures for the establishment of the train sub-model and wheel–rail interaction sub-model in ANSYS can be found in Reference [37].

With the developed programs, the dynamic characteristics of the CRH3 train–CRTS III slab track–subgrade coupled system for the conventional CRTS III slab track without track irregularity (Case 1), the vibration-reduction CRTS III slab track without track irregularity (Case 2), the conventional CRTS III slab track with random track irregularity (Case 3), and the vibration-reduction CRTS III slab track with random track irregularity (Case 4) are simulated, respectively. The largest dynamic responses of the accelerations of the car body, rail, slab, and concrete base, the positive and negative bending moments of rail, the compressive and tension forces of fastener, and the positive and negative bending stresses of slab and concrete base, which are calculated, respectively, using ANSYS^®^ and MATLAB^®^, are listed in Table 3.

It can be seen from Table 3 that there are almost no differences between the largest dynamic responses of the coupled system calculated using ANSYS^®^ and MATLAB^®^, and the maximum difference is less than 0.6% for all items and cases, except for the rail acceleration under random track irregularity (Cases 18 and 24 in Table 4). Because the frequency of rail acceleration is very high under random track irregularity, and the influence factors for high-frequency vibrations are very complex, it is reasonable and acceptable that the difference for the largest rail acceleration calculated using ANSYS^®^ and MATLAB^®^ under random track irregularity be less than 5%. Thus, the calculated results of the coupled system for different cases are reliable and correct.

## 6. Case Study

To study the influence of vehicle number on the dynamic characteristics of the train–CRTS III slab track–subgrade coupled system, the dynamic characteristics of the coupled system with different vehicle numbers, track types, and track irregularity are theoretically studied and compared.

Six types of vehicle numbers, the values of which are 1, 2, 3, 4, 6, and 8, are considered. Two types of tracks, a conventional CRTS III slab track and a vibration-reduction CRTS III slab track are considered. Two types of track irregularities, with random track irregularity and without track irregularity, are considered. The detailed calculation parameters for the loads of Cases 1–24 are listed in Table 4.

The simulations were carried out using a personal computer with an I7 4820k CPU and 64 GB RAM, and the simulation time for each case was about 0.5 h.

### 6.1. Calculation Results

The calculated largest dynamic responses of the accelerations of car body, rail, slab, and concrete base, the positive and negative bending moments of rail, the compressive and tension forces of fastener, the positive and negative bending stresses of slab, and the concrete base for Cases 1–6, 7–12, 13–18, and 19–24 are listed in Table 5, Table 6, Table 7 and Table 8, respectively.

The relation between vehicle number and accelerations of the car body, rail, slab, and concrete base, positive and negative bending moments of rail, compressive and tension forces of fastener, positive and negative bending stresses of slab, and positive and negative bending stresses of concrete base are shown in Figure 5, Figure 6, Figure 7, Figure 8, Figure 9, Figure 10, Figure 11, Figure 12, Figure 13, Figure 14, Figure 15 and Figure 16, respectively.

### 6.2. Results Discussion

From Table 5, Table 6, Table 7 and Table 8 and Figure 5, Figure 6, Figure 7, Figure 8, Figure 9, Figure 10, Figure 11, Figure 12, Figure 13, Figure 14, Figure 15 and Figure 16, it can be seen that the largest dynamic responses of the coupled system for all items are no longer changed when the vehicle number exceeds three, and three vehicles are adequate to guarantee the simulation precision to investigate the dynamic responses of the coupled system.

**Figure 5:**
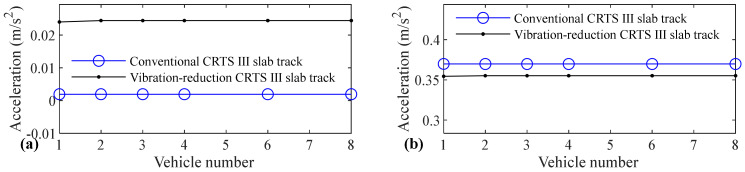
Relation between vehicle number and acceleration of car body: (**a**) without and (**b**) with random track irregularity.

From Figure 5, it is shown that the acceleration of the car body has almost no relation with the vehicle number, whether it is a conventional CRTS III slab track or the vibration-reduction CRTS III slab track, or whether the track is a smooth track condition or a random track irregularity condition. Only one vehicle is needed to study the vehicle dynamics considering the coupling effect between a moving high-speed train and the CRTS III slab track on the subgrade.

**Figure 6:**
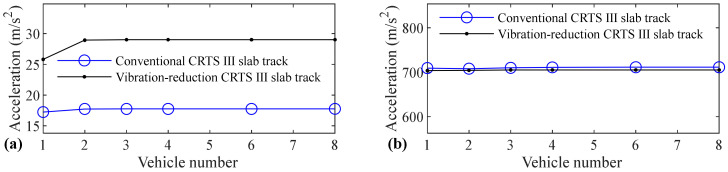
Relation between vehicle number and acceleration of rail: (**a**) without and (**b**) with random track irregularity.

The rail acceleration has some relation with the vehicle number for the vibration-reduction CRTS III slab track without track irregularity, as shown in Figure 6. When the vehicle number increases from one to two, the rail acceleration increases by 12.1%. From Figure 6, we can further see that the rail acceleration has little relation with the vehicle number for the conventional CRTS III slab track without track irregularity, the conventional and vibration-reduction CRTS III slab tracks with random track irregularity, and the rail acceleration increases by 2.9%, −0.2%, and 0.1%, respectively, when the vehicle number increases from one to two. To research the rail acceleration, only one vehicle is needed for the conventional CRTS III slab tracks without track irregularity and with random track irregularity, and the vibration-reduction CRTS III slab track with random track irregularity. However, two vehicles are required for the vibration-reduction CRTS III slab track without track irregularity.

From Table 7, it can be seen that the acceleration of the rail does not increase monotonically with an increase in vehicle numbers, it decreases slightly from 709.289 m/s^2^ to 707.879 m/s^2^ when the vehicle number increases from one to two. The first reason lies in the spatial continuity of the rail vibration, and there is a random error triggered by the finite element dispersion. The second reason may be the inevitable error of the numerical integral solution. The third reason is the fact that the movement of the rail is not always going down, and the rail has a small amplitude upward movement when the rail node is several meters away from the loading position; therefore, rail acceleration does not always increase monotonically with an increase in vehicle number.

**Figure 7:**
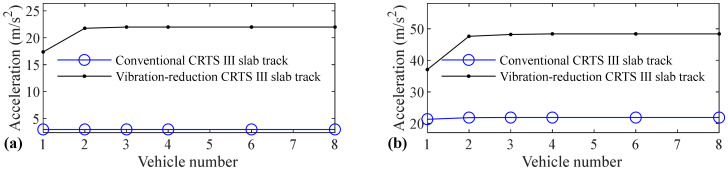
Relation between vehicle number and acceleration of slab: (**a**) without and (**b**) with random track irregularity.

It can be seen from Figure 7 that the vehicle number has a negligible and significant influence on the acceleration of the slab for the conventional and vibration-reduction CRTS III slab tracks, respectively. When the vehicle number increases from one to two, the slab acceleration increases by 25.4% and 28.4% for the vibration-reduction CRTS III slab tracks without track irregularity and with random track irregularity, respectively. When the vehicle number increases from two to three, the increasing of the slab acceleration is below 1.5% for all four cases mentioned above. One vehicle, and two–three vehicles are, respectively, needed for conventional and vibration-reduction CRTS III slab tracks to research the slab acceleration.

**Figure 8:**
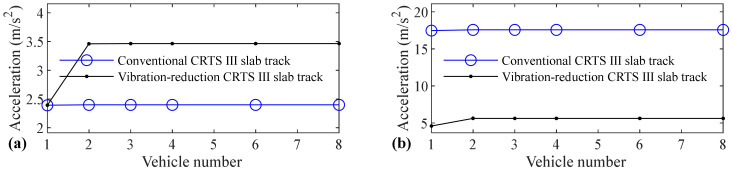
Relation between vehicle number and acceleration of concrete base: (**a**) without and (**b**) with random track irregularity.

As can be clearly observed from Figure 8, vehicle number has a negligible and large influence on the acceleration of the concrete base for conventional and vibration-reduction CRTS III slab tracks, respectively. When the vehicle number increases from one to two, the concrete base acceleration increases by 44.7% and 22.3% for the vibration-reduction CRTS III slab tracks without track irregularity and with random track irregularity, respectively. When the vehicle number increases from two to three, the increase is below 0.2%. One vehicle and two vehicles are, respectively, needed for the conventional and vibration-reduction CRTS III slab tracks to research the concrete base acceleration.

**Figure 9:**
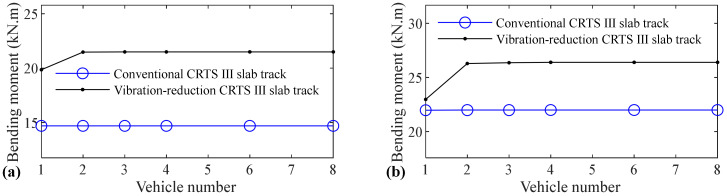
Relation between vehicle number and positive bending moment of rail: (**a**) without and (**b**) with random track irregularity.

From Figure 9, it can be concluded that vehicle number has a negligible influence on the positive bending moment of rail for the conventional and some influence on the vibration-reduction CRTS III slab tracks, respectively. When the vehicle number increases from one to two, the positive bending moment of the rail increases by 8.1% and 14.4% for the vibration-reduction CRTS III slab tracks without track irregularity and with random track irregularity, respectively. When the vehicle number increases from two to three, the increase is below 0.3%. One vehicle, and two vehicles are, respectively, needed for the conventional and vibration-reduction CRTS III slab tracks to study the positive bending moment of rail.

**Figure 10:**
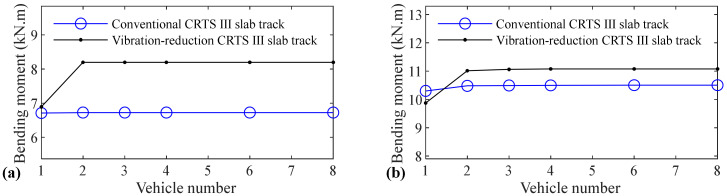
Relation between vehicle number and negative bending moment of rail: **(a)** without and **(b)** with random track irregularity.

Vehicle number has a negligible influence on the negative bending moment of rail for the conventional and some influence on the vibration-reduction CRTS III slab tracks, respectively, and the influence law of the vehicle number on the negative bending moment of rail is similar to that on the positive bending moment of rail, which can be concluded by comparing Figure 10 with Figure 9. When the vehicle number increases from one to two, the negative bending moment of the rail increases by 19.0% and 11.6% for the vibration-reduction CRTS III slab tracks without track irregularity and with random track irregularity, respectively. When the vehicle number increases from two to three, the increase is below 0.5%. One vehicle and two vehicles are, respectively, required for the conventional and vibration-reduction CRTS III slab tracks to research the negative bending moment of rail.

**Figure 11:**
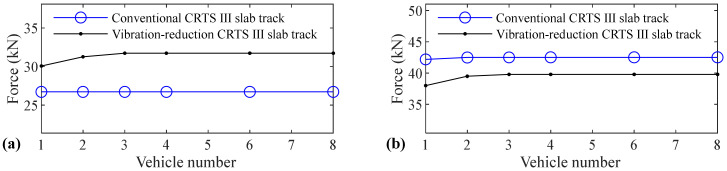
Relation between vehicle number and compressive force of fastener: (**a**) without and (**b**) with random track irregularity.

From Figure 11, it can be seen that vehicle number has a negligible influence on the compressive force of fastener for the conventional and a small influence on the vibration-reduction CRTS III slab tracks, respectively. When the vehicle number increases from one to two, the compressive force of the fastener increases by 4.0% and 3.9% for the vibration-reduction CRTS III slab tracks without track irregularity and with random track irregularity, respectively. When the vehicle number increases from two to three, the increase is below 1.6%. Only one vehicle is needed if the simulation precision is not strictly required, and two–three vehicles should be considered if the simulation precision is required to research the compressive force of the fastener.

**Figure 12:**
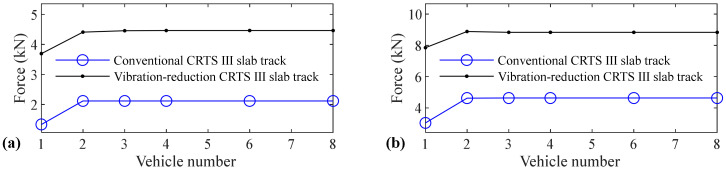
Relation between vehicle number and tension force of fastener: (**a**) without and (**b**) with random track irregularity.

It is apparent that the influence of vehicle number on the tension force of the fastener is much larger than the compressive force of the fastener, which can be seen by comparing Figure 12 with Figure 11. When the vehicle number increases from one to two, the increases in the fastener tension forces are 58.6%, 52.2%, 19.4%, are 13.2% respectively, for the conventional CRTS III slab tracks without track irregularity and with random track irregularity and the vibration-reduction CRTS III slab tracks without track irregularity and with random track irregularity. The increase in the fastener tension force is below 1.3% for all four cases above when the vehicle number increases from two to three. We can further deduce from Figure 12 that vehicle number has a larger influence on the tension force of the fastener for the conventional CRTS III slab track than that for the vibration-reduction CRTS III slab track, which is in contrast to the influence law of vehicle number on the bending moment of the rail in Figure 9 and Figure 10, and the compressive force of the fastener in Figure 11. At least two vehicles are required to study the tension force of the fastener.

**Figure 13:**
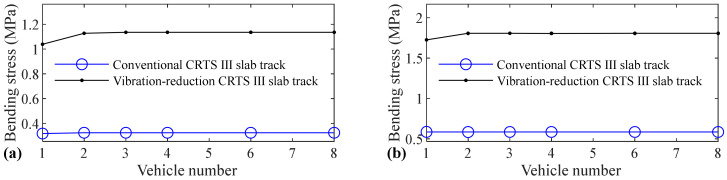
Relation between vehicle number and positive bending stress of slab: (**a**) without and (**b**) with random track irregularity.

It is clear from Figure 13 that vehicle number has a negligible influence on the positive bending stress of slab for the conventional and some influence on the vibration-reduction CRTS III slab tracks, respectively. When the vehicle number increases from one to two, the positive bending stress of the slab increases by 8.6% and 4.7% for the vibration-reduction CRTS III slab tracks without track irregularity and with random track irregularity, respectively. When the vehicle number increases from two to three, the increase is below 0.7%. One vehicle and two vehicles are, respectively, needed for conventional and vibration-reduction CRTS III slab tracks to research the positive bending stress of the slab.

**Figure 14:**
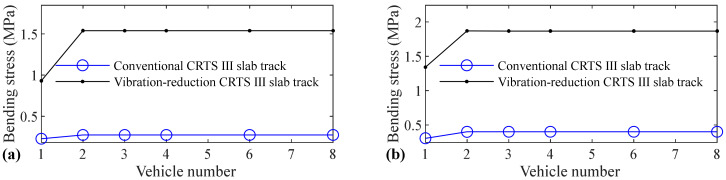
Relation between vehicle number and negative bending stress of slab: (**a**) without and (**b**) with random track irregularity.

By comparing Figure 14 with Figure 13, it is clear that the influence of vehicle number on the negative bending stress of the slab is much larger than the positive bending stress of the slab. The increases are 20.5%, 30.8%, 65.5%, and 39.3%, respectively, for the conventional CRTS III slab tracks without track irregularity and with random track irregularity, the vibration-reduction CRTS III slab tracks without track irregularity and with random track irregularity when the vehicle number increases from one to two. From Figure 14, it is also easy to find that vehicle number has a larger influence on the negative bending stress of the slab for the vibration-reduction CRTS III slab track than that for the conventional CRTS III slab track. Furthermore, we can find from Figure 14 that the increase is below 0.2% for all four cases mentioned above when the vehicle number increases from two to three. Two vehicles are required to study the negative bending stress of slab.

**Figure 15:**
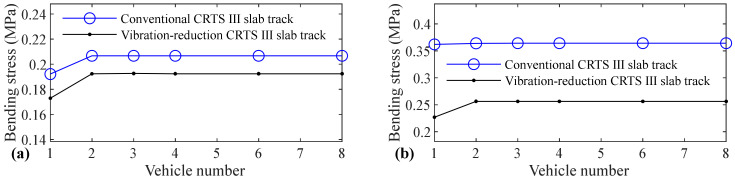
Relation between vehicle number and positive bending stress of concrete base: (**a**) without and (**b**) with random track irregularity.

Figure 15 shows that vehicle number has some influence on the positive bending stress of the concrete base. The increases are 7.6%, 0.6%, 11.3%, and 12.9% for the conventional CRTS III slab tracks without track irregularity and with random track irregularity, the vibration-reduction CRTS III slab tracks without track irregularity and with random track irregularity, respectively, when the vehicle number increases from one to two. When the vehicle number increases from two to three, the increase is below 0.2% for all four cases. Only one vehicle is needed if simulation precision is not strictly required, and two vehicles should be considered if the simulation precision is required to research the positive bending stress of the concrete base.

**Figure 16:**
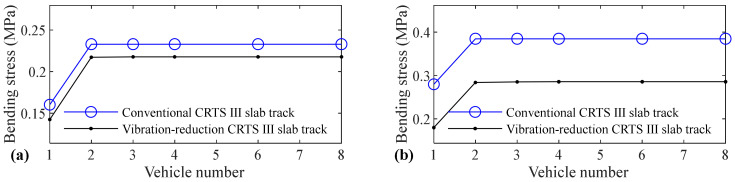
Relation between vehicle number and negative bending stress of concrete base: (**a**) without and (**b**) with random track irregularity.

It can be found by comparing Figure 16 with Figure 15 that the influence of vehicle number on the negative bending stress of the concrete base is much larger than the positive bending stress of the concrete base. The increases are 45.3%, 37.5%, 52.5%, and 57.9%, respectively, for the conventional CRTS III slab tracks without track irregularity and with random track irregularity, the vibration-reduction CRTS III slab tracks without track irregularity and with random track irregularity when the vehicle number increases from one to two. The increase is below 0.7% for all four cases mentioned above when the vehicle number increases from two to three. At least two vehicles are required to study the negative bending stress of the concrete base.

## 7. Conclusions

A high speed train–CRTS III slab track–subgrade coupling dynamic model is established. Using this model, the influence of vehicle number on the dynamic characteristics of train–CRTS III slab track–subgrade coupled system with smooth and random track irregularity conditions for conventional and vibration-reduction CRTS III slab tracks is theoretically studied and analyzed. Some conclusions can be drawn from the calculated results.

- The acceleration of the car body has almost no relation with the vehicle number, and only one vehicle is needed to study vehicle dynamics using the train–CRTS III slab track–subgrade coupled dynamic model.
- For the conventional CRTS III slab track on a subgrade, vehicle number has a negligible influence on the accelerations of rail, slab, and concrete base, the positive and negative bending moments of rail, the compressive force of fastener, and the positive bending stress of slab. However, vehicle number has some influence on the positive bending stress of the concrete base, and has a large influence on the tension force of the fastener, negative bending stresses of slab and concrete base. Only one vehicle is needed to study the track dynamics for the conventional CRTS III slab track on a subgrade without considering the tension force of the fastener, the negative bending stresses of slab and concrete base; otherwise, two or three vehicles are needed.
- The number of vehicles has some influence on the dynamic responses of all track components for the vibration-reduction CRTS III slab track on the subgrade. At least two vehicles are required to research the track dynamics for the vibration-reduction CRTS III slab track on a subgrade.

## Figures and Tables

**Table 1:** The vehicle parameters of CRH3 train.

Item	Values	Item	Values
Car body mass [kg]	21,200	Stiffness of primary suspension [kN/m]	1040
Bogie mass [kg]	1700	Damping of secondary suspension [kN∙s/m]	35
Wheel mass [kg]	1100	Stiffness of secondary suspension [kN/m]	400
Pitch inertia of car body [kg∙m^2^]	1,370,000	Wheelbase [m]	2.5
Pitch inertia of bogie [kg∙m^2^]	3600	Distance between two centers of bogie [m]	17.375
Damping of primary suspension [kN∙s/m]	35	Vehicle length [m]	26.3

**Table 2:** Calculation parameters of the CRTS III slab track-subgrade sub-model.

Parameters	Value	Parameters	Value
Elastic modulus of rail [Pa]	2.1 × 10^11^	Width of slab [m]	2.5
Density of rail [kg/m^3^]	7800	Density of slab and concrete base [kg/m^3^]	2500
Area of rail [m^2^]	7.745 × 10^−^^3^	Contact stiffness between slab and concrete base [N/m]	1.31 × 10^8^
Moment of inertia of rail [m^4^]	3.217 × 10^−5^	Contact damping between slab and concrete base [N∙s/m]	1.16 × 10^4^
Space of fastener [m]	0.63	Elastic modulus of concrete base [Pa]	3.25 × 10^10^
Stiffness of fastener [kN/mm]	40	Thickness of concrete base [m]	0.3
Damping of fastener [kN∙s/m]	20	Width of concrete base [m]	3.1
Elastic modulus of slab [Pa]	3.6 × 10^10^	Contact stiffness between concrete base and subgrade [N/m]	3.10 × 10^7^
Thickness of slab [m]	0.29	Contact damping between concrete base and subgrade [N∙s/m]	1.36 × 10^4^

**Table 3:** Comparison of the largest dynamic responses of the coupled system calculated using ANSYS and MATLAB for different cases.

Item		Case 1		Case 2		Case 3		Case 4	
		ANSYS	MATLAB	ANSYS	MATLAB	ANSYS	MATLAB	ANSYS	MATLAB
Acceleration (m/s^2^)	Car body	0.002	0.002	0.024	0.024	0.37	0.37	0.355	0.355
	Rail	17.673	17.755	28.915	29.015	681.627	711.183	691.271	705.129
	Slab	2.969	2.97	21.98	21.98	21.748	21.862	48.31	48.38
	Concrete base	2.397	2.397	3.464	3.464	17.556	17.565	5.633	5.635
Bending moment of rail (kN∙m)	Positive	14.698	14.698	21.485	21.485	21.986	21.991	26.392	26.394
	Negative	6.725	6.725	8.194	8.194	10.507	10.505	11.076	11.077
Fastener force(kN)	Compressive	26.719	26.719	31.741	31.741	42.515	42.514	39.783	39.783
	Tension	2.116	2.116	4.461	4.461	4.633	4.633	8.833	8.833
Bending stress of slab (MPa)	Positive	0.324	0.324	1.135	1.135	0.585	0.585	1.806	1.806
	Negative	0.274	0.274	1.54	1.54	0.401	0.401	1.868	1.868
Bending stress of concrete base (MPa)	Positive	0.207	0.207	0.192	0.192	0.364	0.364	0.256	0.256
	Negative	0.233	0.233	0.218	0.218	0.385	0.385	0.286	0.286

**Table 4:** Calculation parameters for load Cases 1–24.

Case	Vehicle Number	CRTS III Slab Track Type	Track Irregularity Type	Case	Vehicl Number	CRTS III Slab Track Type	Track Irregularity Type
1	1	conventional	without	13	1	conventional	random track irregularity
2	2	conventional	without	14	2	conventional	random track irregularity
3	3	conventional	without	15	3	conventional	random track irregularity
4	4	conventional	without	16	4	conventional	random track irregularity
5	6	conventional	without	17	6	conventional	random track irregularity
6	8	conventional	without	18	8	conventional	random track irregularity
7	1	vibration-reduction	without	19	1	vibration-reduction	random track irregularity
8	2	vibration-reduction	without	20	2	vibration-reduction	random track irregularity
9	3	vibration-reduction	without	21	3	vibration-reduction	random track irregularity
10	4	vibration-reduction	without	22	4	vibration-reduction	random track irregularity
11	6	vibration-reduction	without	23	6	vibration-reduction	random track irregularity
12	8	vibration-reduction	without	24	8	vibration-reduction	random track irregularity

**Table 5:** Largest dynamic responses for Cases 1–6.

Case	1	2	3	4	5	6
Acceleration of car body (m/s^2^)	0.002	0.002	0.002	0.002	0.002	0.002
Acceleration of rail (m/s^2^)	17.246	17.743	17.749	17.750	17.753	17.755
Acceleration of slab (m/s^2^)	2.969	2.970	2.970	2.970	2.970	2.970
Acceleration of concrete base (m/s^2^)	2.390	2.397	2.397	2.397	2.397	2.397
Positive bending moment of rail (kN∙m)	14.696	14.698	14.698	14.698	14.698	14.698
Negative bending moment of rail (kN∙m)	6.711	6.725	6.725	6.725	6.725	6.725
Compressive force of fastener (kN)	26.722	26.719	26.719	26.719	26.719	26.719
Tension force of fastener (kN)	1.334	2.116	2.116	2.116	2.116	2.116
Positive bending stress of slab (MPa)	0.318	0.324	0.324	0.324	0.324	0.324
Negative bending stress of slab (MPa)	0.227	0.274	0.274	0.274	0.274	0.274
Positive bending stress of concrete base (MPa)	0.192	0.207	0.207	0.207	0.207	0.207
Negative bending stress of concrete base (MPa)	0.160	0.233	0.233	0.233	0.233	0.233

**Table 6:** Largest dynamic responses for Cases 7–12.

Case	7	8	9	10	11	12
Acceleration of car body (m/s^2^)	0.024	0.024	0.024	0.024	0.024	0.024
Acceleration of rail (m/s^2^)	25.801	28.930	29.020	29.015	29.015	29.015
Acceleration of slab (m/s^2^)	17.363	21.770	21.980	21.980	21.980	21.980
Acceleration of concrete base (m/s^2^)	2.393	3.461	3.464	3.464	3.464	3.464
Positive bending moment of rail (kN∙m)	19.859	21.475	21.487	21.485	21.485	21.485
Negative bending moment of rail (kN∙m)	6.891	8.198	8.194	8.194	8.194	8.194
Compressive force of fastener (kN)	30.069	31.269	31.736	31.741	31.741	31.741
Tension force of fastener (kN)	3.694	4.410	4.457	4.461	4.461	4.461
Positive bending stress of slab (MPa)	1.039	1.128	1.135	1.135	1.135	1.135
Negative bending stress of slab (MPa)	0.930	1.540	1.540	1.540	1.540	1.540
Positive bending stress of concrete base (MPa)	0.173	0.192	0.193	0.192	0.192	0.192
Negative bending stress of concrete base (MPa)	0.143	0.217	0.218	0.218	0.218	0.218

**Table 7:** Largest dynamic responses for Cases 13–18.

Case	13	14	15	16	17	18
Acceleration of car body (m/s^2^)	0.370	0.370	0.370	0.370	0.370	0.370
Acceleration of rail (m/s^2^)	709.289	707.879	709.844	710.845	711.190	711.183
Acceleration of slab (m/s^2^)	21.337	21.835	21.862	21.863	21.862	21.862
Acceleration of concrete base (m/s^2^)	17.469	17.566	17.565	17.565	17.565	17.565
Positive bending moment of rail (kN∙m)	21.966	21.983	21.983	21.990	21.990	21.991
Negative bending moment of rail (kN∙m)	10.301	10.480	10.493	10.499	10.504	10.505
Compressive force of fastener (kN)	42.183	42.487	42.499	42.511	42.512	42.514
Tension force of fastener (kN)	3.042	4.630	4.632	4.633	4.633	4.633
Positive bending stress of slab (MPa)	0.586	0.584	0.585	0.585	0.585	0.585
Negative bending stress of slab (MPa)	0.306	0.400	0.401	0.401	0.401	0.401
Positive bending stress of concrete base (MPa)	0.362	0.364	0.364	0.364	0.364	0.364
Negative bending stress of concrete base (MPa)	0.280	0.385	0.385	0.385	0.385	0.385

**Table 8:** Largest dynamic responses for Cases 19–24.

Case	19	20	21	22	23	24
Acceleration of car body (m/s^2^)	0.354	0.355	0.355	0.355	0.355	0.355
Acceleration of rail (m/s^2^)	703.467	704.228	705.093	705.119	705.129	705.129
Acceleration of slab (m/s^2^)	37.096	47.619	48.170	48.375	48.380	48.380
Acceleration of concrete base (m/s^2^)	4.610	5.638	5.635	5.635	5.635	5.635
Positive bending moment of rail (kN∙m)	22.971	26.284	26.356	26.394	26.394	26.394
Negative bending moment of rail (kN∙m)	9.875	11.016	11.060	11.077	11.077	11.077
Compressive force of fastener (kN)	38.002	39.484	39.777	39.776	39.782	39.783
Tension force of fastener (kN)	7.845	8.880	8.834	8.833	8.833	8.833
Positive bending stress of slab (MPa)	1.726	1.806	1.806	1.806	1.806	1.806
Negative bending stress of slab (MPa)	1.342	1.870	1.868	1.868	1.868	1.868
Positive bending stress of concrete base (MPa)	0.227	0.256	0.256	0.256	0.256	0.256
Negative bending stress of concrete base (MPa)	0.180	0.284	0.285	0.286	0.286	0.286

## Data Availability

Not applicable.
